# Comparison of Energy Expenditure Assessed Using Wrist- and Hip-Worn ActiGraph GT3X in Free-Living Conditions in Young and Older Adults

**DOI:** 10.3389/fmed.2021.696968

**Published:** 2021-08-31

**Authors:** Amine Guediri, Louise Robin, Justine Lacroix, Timothee Aubourg, Nicolas Vuillerme, Stephane Mandigout

**Affiliations:** ^1^University of Limoges, HAVAE, EA 6310, Limoges, France; ^2^Univ. Grenoble Alpes, AGEIS, Grenoble, France; ^3^LabCom Telecom4Health, Orange Labs and Univ. Grenoble Alpes, CNRS, Inria, Grenoble INP-UGA, Grenoble, France; ^4^Institut Universitaire de France, Paris, France

**Keywords:** activity tracker, energy expenditure, location, free living, older

## Abstract

The World Health Organization has presented their recommendations for energy expenditure to improve public health. Activity trackers do represent a modern solution for measuring physical activity, particularly in terms of steps/day and energy expended in physical activity (active energy expenditure). According to the manufacturer's instructions, these activity trackers can be placed on different body locations, mostly at the wrist and the hip, in an undifferentiated manner. The objective of this study was to compare the absolute error rate of active energy expenditure measured by a wrist-worn and hip-worn ActiGraph GT3X+ over a 24-h period in free-living conditions in young and older adults. Over the period of a 24-h period, 22 young adults and 22 older adults were asked to wear two ActiGraph GT3X+ at two different body locations recommended by the manufacturer, namely one around the wrist and one above the hip. Freedson algorithm was applied for data analysis. For both groups, the absolute error rate tended to decrease from 1,252 to 43% for older adults and from 408 to 46% for young participants with higher energy expenditure. Interestingly, for both young and older adults, the wrist-worn ActiGraph provided a significantly higher values of active energy expenditure (943 ± 264 cal/min) than the hip-worn (288 ± 181 cal/min). Taken together, these results suggest that caution is needed when using active energy expenditure as an activity tracker-based metric to quantify physical activity.

## Introduction

The daily estimation of energy expenditure (EE) has been a major issue in recent years in the effort to improve public health (1). EE has been used as a variable to assess physical activity (PA) by activity trackers (AT) and a parameter in presenting recommendations for PA (2). Total energy expenditure (TEE), representing the daily energy required by the organism, is determined by the sum of three components: basal energy expenditure, diet-induced thermogenesis and PA (i.e., active energy expenditure, AEE) (3). The World Health Organization and other institutions have presented their recommendations on PA: Adults should do at least 150–300 min of moderate-intensity aerobic physical activity throughout the week, for substantial health benefits. A moderate intensity physical activity is between 3 and 6 times the energy expenditure at rest. At this point, however, the accuracy of EE estimation in real life situations seems rather difficult to obtain. This challenge can stem from various non-exclusive factors, including, amongst others, the placement of the AT on the body, which is linked to the characteristic of the sensor (pedometer, accelerometer, etc.) or users age and/or ability to walk. There are several methods used for EE measurement, such as indirect and direct calorimetry, bioelectrical impedance, doubly labeled water, predictive equations and questionnaires. Doubly labeled water and direct calorimetry are the reference methods of assessment for TEE. In free living conditions, we can assess TEE only by doubly labeled water (4). Nevertheless, this method is expensive and difficult to implement in practice. As a substitute, wearable sensors have been developed to evaluate EE, and they have been increasingly explored in several research fields (2). However, these alternative methods can provide assessment of AEE only.

Currently, the most used technology is the accelerometer, with two main placements on the body, wrist and hip (5). Independently of placement, the sensors should provide the same results for AEE, step counts and all collected parameters to allow individuals to assess their PA and to follow the recommendations.

The ActiGraph GT3X+ is the pinnacle of scientific accelerometers (ActiGraph LLC, Pensacola, FL, USA). It's discreet, and the sensitive triaxial accelerometers can hold high-resolution, raw, unfiltered acceleration signals for an extended period of time. The ActiGraph monitor has been extensively used for different purposes: as validation for the evaluation of PA (EE, step counts) in healthy (6, 7) or pathological populations such as post-stroke individuals (8, 9) and in comparison, with other ATs (10–14). Actigraph monitor is further considered as a gold standard in some studies (15, 16).

In a recent systematic review by Migueles et al. (17), 235 published articles assessing sedentary time, PA, EE or sleep using the ActiGraph GT3X/+ were included. Of them, 103 were conducted in young adults and 51 in older adults (validation/calibration studies). These authors concluded that caution is necessary with regard to the accuracy/reliability of the ActiGraph GT3X/+ in estimating the AEE in free living conditions. They further identified two main factors that have to be taken into consideration, (1) the location of this activity tracker on the body (wrist and hip) and (2) the age of the users. In addition, as concluded by the authors themselves (17), there are only a few studies (18–24) directly comparing two placement sites using the GT3X/+ and they have consistently shown more accurate classification of sedentary time and PA intensity as well as estimates of physical active energy expenditure when the accelerometer was worn on the hip compared to the wrist. The hip has been the most commonly used placement for studies in older adults (17). For young healthy individuals (40 participants, mean age: 35.8 ± 12.1 years), Ellis et al. (18) reported that the ActiGraph placed on the hip had a better estimation for AEE than when placed on the wrist while performing walking and household activities in a lab. However, for the same studied population, Lee and Tse (25) found that wrist-worn Actiwatch 2 and ActiGraph wGT3X-BT were strongly correlated in PA assessment to energy expenditure measured by indirect calorimetry while running at different speeds on a treadmill. In another study (26), the authors recommend wearing Actigraph on the hip to assess step rates in older adults.

Some research have focused on the estimation of the absolute error rate (AER) in order to better analyze accuracy depending to the position or type of AT (27–30). Interestingly, this approach allows for the evaluation of whether wrist-hip discrepancies are due to an AT or individual factor.

In a recent study (31), we compared the difference in step counts between the hip-worn and wrist-worn AT in young and older adults. We showed that the difference between both measurements tended to decrease for longer distances. In the present work, we are going to use the same data collected on the step count project with a focus on the AEE. To the best of our knowledge, no research has looked at the relationship between the percentage of absolute error between the AEE of the two most used AT positions (hip and wrist) and the user's age when using the ActiGraph GT3X. The objective of this work was to examine the AER of AEE measured by the wrist-orn and hip-worn ActiGraph GT3X in young and older persons over a 24-h period in free-living situations.

## Materials and Methods

This study uses the data collected from the same population during our study of step count (31) recently published on “Frontiers in Medicine.” We mention also that this is the same method part already published (31). In the present paper, we specifically and solely focus on AEE.

### Study Population

Our exploratory study's population was aged between 18 and 85 years, without medical contraindications, who volunteered to participate. The exclusion criteria were any cardiovascular pathologies or mobility issues. The sample was divided into two groups: a group of participants aged 18–45 years and a group of subjects aged 70–85 years. The protocol was approved by the Comité d'Ethique pour les Recherches Non Interventionnelles (Ethics Committee for Non-Interventional Research) (CERNI) of the Grenoble-Alpes University, France. All subjects gave their written informed consent in accordance with the Declaration of Helsinki.

### Materials

The material requirements for the study were two ActiGraph GT3X+ accelerometers (ActiGraph Pensacola, FL, USA, www.actigraphcorp.com). These triaxial accelerometers are used to record step counts along with various PA data such as active energy expenditure. Accelerometer data were collected at a frequency of 80 Hz and aggregated to 60-second periods for analyses. Following the manufacturer's guidelines (32), a low frequency extension (LFE) filter was used to increase the device's sensitivity and detect low-frequency accelerations (i.e., slow walking). The Freedson algorithm was applied for data analysis. We indicated the placement of the AT on the software at the beginning of every data analysis.

### Experimental Design

This study was designed to record the PA of a sample population in a free-living situation for 24 h. The two ActiGraph monitors were positioned as follows: one ActiGraph GT3X+ at the hip (in the center of the pelvis) and a second one on the non-dominant wrist. Participants were asked to remove the device before showering and for aquatic activities. To compare accelerometer data according to the body location, we limited the data from all devices to the actual wearing time for both devices. The AT was placed in the morning (between 8 and 11 am) and was picked up the next day at the same time. The registration period was programmed using the manufacturer's ActiLife software v 6.13.3 (www.actigraphcorp.com/actilife/). A 24-h recording period allowed us to avoid the risk of human failure (weariness, forgetfulness, etc.). After verification by an investigator, the records appeared to be correct and usable.

The parameter used in this study was AEE. The equipment was activated before placing it on the previously described body locations. The minimum required to record duration of accelerometer data to be included in the analysis was 24 h for both ATs. After the 24 h of recording, the subject was asked to return the equipment to allow the practitioner to transfer it using ActiLife and reset the devices for new use.

### Statistical Analysis

The data are presented in the form of means and standard deviations (SD).

**Figure 1:**
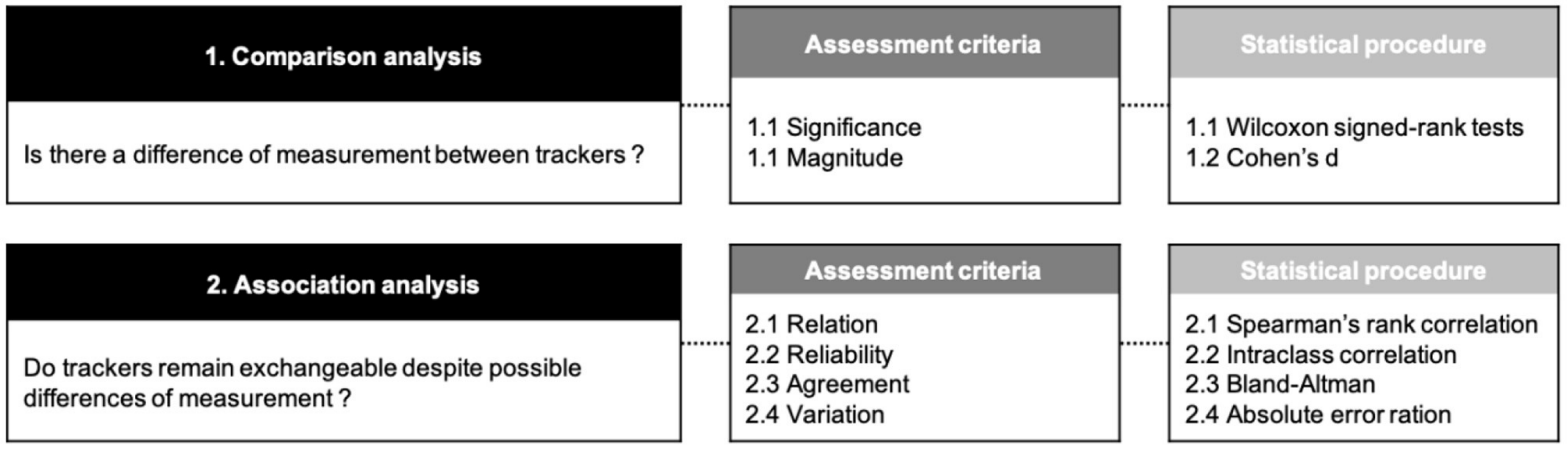
Synthesis diagram of the statistical treatment.

To compare these data in free-living conditions in young and older adults according to the AT location on the body (i.e., hip vs. wrist), statistical tests of comparison were selected by testing AEE data for normal distribution using the Shapiro-Wilk test. As the dependent variables did not conform to a Gaussian distribution, non-parametric comparative tests were chosen for the statistical analysis process. These statistical tests were then performed in two successive steps, as described in Figure 1.

#### Step 1: Comparison Analysis

We compared the differences between AEE provided by the wrist-worn and hip-worn AT using two assessment criteria, namely (1) the significance and (2) the effect size of these differences. Significant differences were assessed by means of non-parametric Wilcoxon signed-rank tests. Effect sizes, also known as magnitude, were obtained using Cohen's d. This coefficient was calculated as a ratio of the mean difference divided by the mean standard deviation in both conditions. Effect sizes were considered small if *d* < 0.5, medium if 0.5 ≤ *d* < 0.8 and large with *d* ≥ 0.8. We completed this statistical procedure by comparing the differences in measurement between hip-worn and wrist-worn ATs according to the age category of the participants, using Wilcoxon signed-rank tests.

#### Step 2: Association Analysis

The results from step 1 were complemented with an additional analysis to determine whether ATs could remain exchangeable despite potential differences of measurement, and if so, to what extent. For this purpose, four assessment criteria were used; (1) relation, (2) reliability, (3) agreement and (4) variation. The relationship between AEE measrued by the wrist-worn and hip-worn AT was calculated by means of Spearman's rank correlation coefficient rho. Reliability was measured by means of the intraclass correlation coefficient (ICC). An ICC value between 0.00 and 0.40 was considered poor, 0.40 and 0.59 fair, 0.60 and 0.74 good, and 0.75 and 1.00 excellent. The obtained scores were reported in Bland-Altman plots to visualize the agreement between the wrist-worn and hip-worn AT. Finally, for comparison purposes, we assumed the hip location could be used as reference to study errors of measurement. To this end, we assessed the variation of the error measurement generated by the wrist-worn AT according to the AEE provided by the hip-worn AT. The absolute error ratio (AER) for each estimated parameter was hence determined relative to the hip-worn AT as follows:

$$\begin{array}{l} \text{AER}=(\text{value at the wrist}-\text{value at the hip}/\\ \text{ value at the hip})\;*100 \end{array}$$

The level of significance was set as *p* < 0.05 in all statistical tests. All statistical calculations were completed using the R software environment (version 3.1.0; R Foundation for Statistical Computing, Vienna, Austria).

## Results

A total of 44 volunteers participated in this study (22 per group).

In the young group, there were 14 males and eight females. Of them, 18 were right-handed and four left-handed. The mean age was 27.2 years (SD = 6). The average weight was 72.4 kg (SD = 13). With regard to the height, the participants in this group measured 173.8 cm on average (SD = 7.9).

In the older group, there were eight males and 14 females. Of them, 19 were right-handed and three left-handed. The mean age was 76.6 years (SD = 4.7). The average weight was 65.3 kg (SD = 10.1). With regard to the height, the participants in this group measured 162.8 cm on average (SD = 7.5).

### Comparison Between AEE Provided by the Hip-Worn and the Wrist-Worn Activity Tracker

All results are provided on Table 1. Regarding the overall population, wrist-worn AT AEE values were significantly lower than hip-worn AT AEE values with, respectively, 943 cal/min (SD = 264) vs. 288 cal/min (SD = 181) on average. A significant difference was also observed in the young participants group, with, respectively, 1,034 cal/min (SD = 239) for the wrist-worn AT AEE vs. 367 cal/min (SD = 186) for the hip-worn AT AEE. Interestingly, this difference increased for older participants: the mean AEE provided by the wrist-worn AT was more than four times higher than the value provided by the hip-worn AT, with, respectively, 852 cal/min (SD = 262) vs. 209 cal/min (SD = 140) on average. In addition, Cohen's d demonstrated the large effect size of this phenomenon for the overall population, but also for both young and older participants (*d* > 0.9). Finally, we may note that the reliability assessed by the ICC test was fair, from 0.43 to 0.53 for the three cases.

**Table 1:** AEE Descriptive statistics, comparison, correlation, agreement, and Bland-Altman parameters for the Active energy expenditure (AEE) provided by the wrist-worn and hip-worn AT for all participants and by age group.

	**All**	**Young**	**Old**
Wrist-worn (cal/min) mean (SD)	943 (264)	1,034 (239)	852 (262)
Hip-worn (cal/min) mean (*SD*)	288 (181)	367 (186)	209 (140)
Wilcoxon test: between hip and wrist *Z*-value; *p*-value	5.78; 1.14e-13	4.11; 4.77e-07	4.01; 4.5e-07
Regression coefficient^*^: α; β	749; 0.68	776; 0.70	787; 0.31
Cohen's *d*	2.7	3.2	2.3
ICC (95% CI)	0.43 (0.16–0.64)	0.53 (0.14–0.77)	0.48 (0.09–0.87)
Spearman's correlation between hip and wrist	0.48 *p* < 0.001	0.25 *p* = 0.27	0.57 *p* < 0.001
Spearman's correlation between AER and hip-worn	−0.84 *p* < 0.001	−0.83 *p* < 0.001	−0.87 *p* < 0.001
Mean of differences (95% limits of agreement)	655 (18, 1,129)	667 (258, 1,076)	643 (103, 1,183)
AER mean (SD)	351% (292)	231% (122)	470% (361)
Mann-Whitney test for the difference between young and older participants in AER: *Z*-value; *p*-value		−1,99; 0.04	

* *From the regression equation: Mwrist = α + β.Mhip, where Mwrist is the wrist-worn measurements variable and Mhip is the hip-worn measurements variable*.

### Association Between AEE Provided by the Hip-Worn and the Wrist-Worn Activity Tracker

**Figure 2:**
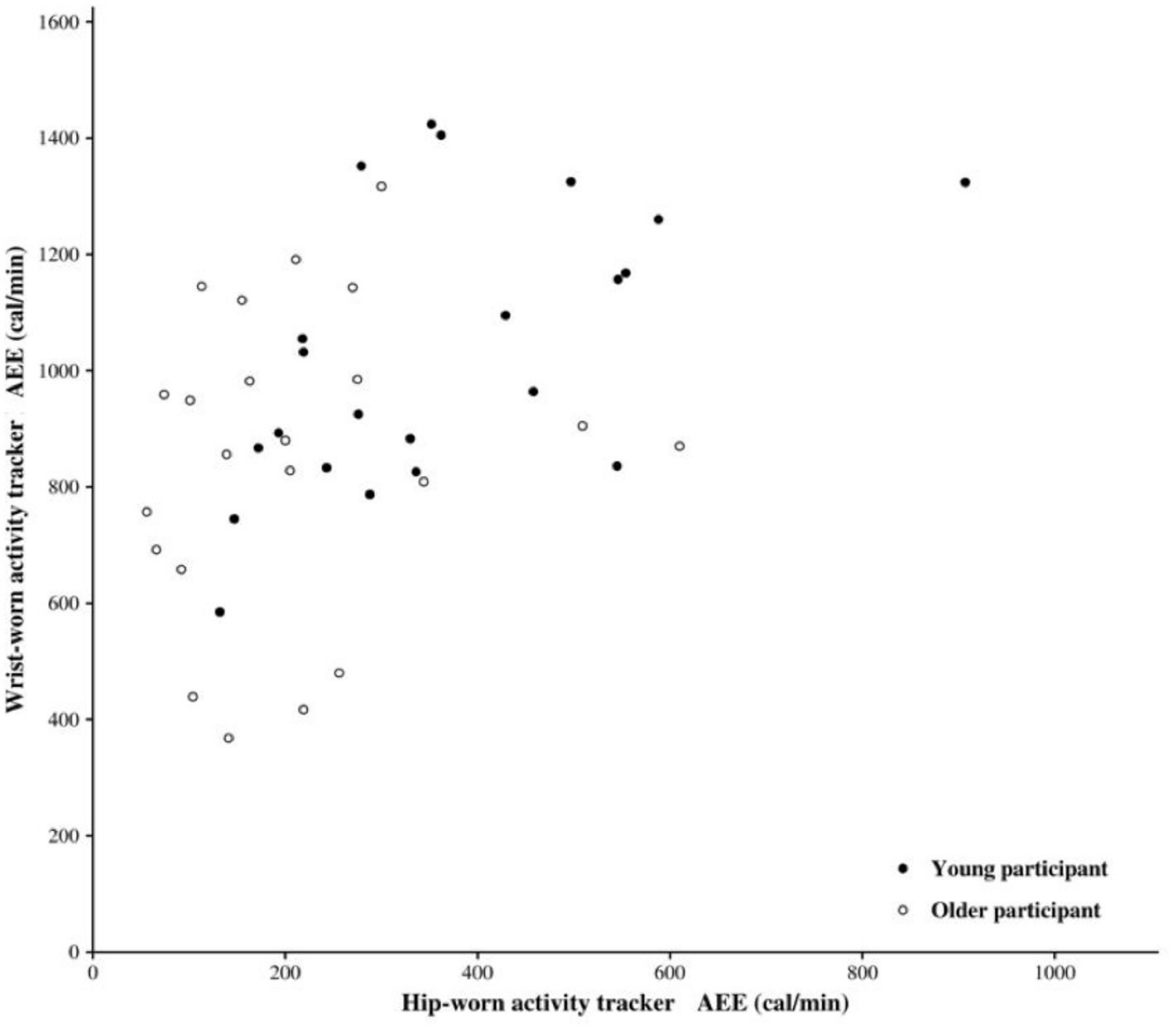
Association between active energy expenditure (AEE) measured by hip-worn and wrist-worn activity tracker.

In the overall population and older participants, there was a significant positive correlation between AEE valesu measured by the wrist-worn AT and those measured by the hip-worn AT (spearman's rho = 0.48 and 0.57, respectively, *p* < 0.001). No correlation was found for the young participants (spearman's rho = 0.25, *p* = 0.27) (Figure 2).

**Figure 3:**
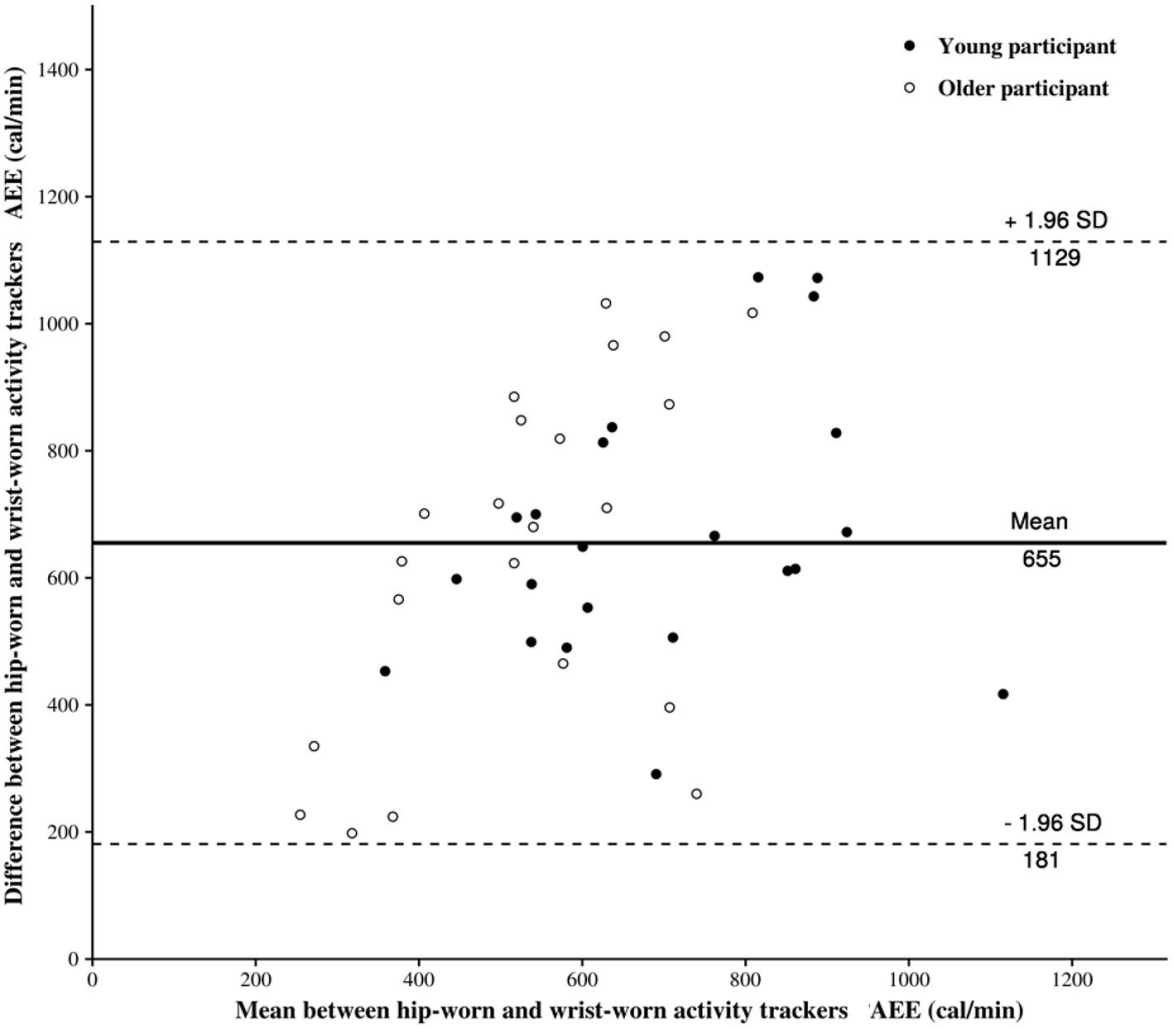
The Bland-Altman plot for the active energy expenditure (AEE) provided by hip-worn and wrist-worn activity trackers.

The Bland-Altman plot for the AEE provided by both AT locations on the body is provided in Figure 3. The estimated bias, i.e., the mean of the differences between the wrist-worn and hip-worn AT measures, is 655, as shown in Table 1. This result suggests that when compared to the hip-worn AT, the wrist-worn AT tends to overestimate the AEE.

### Variation of the Error Measurement

We calculated the error rate generated by the AT's position on the body in order to account for the chance that this error would reduce if a particular threshold was reached.

**Figure 4:**
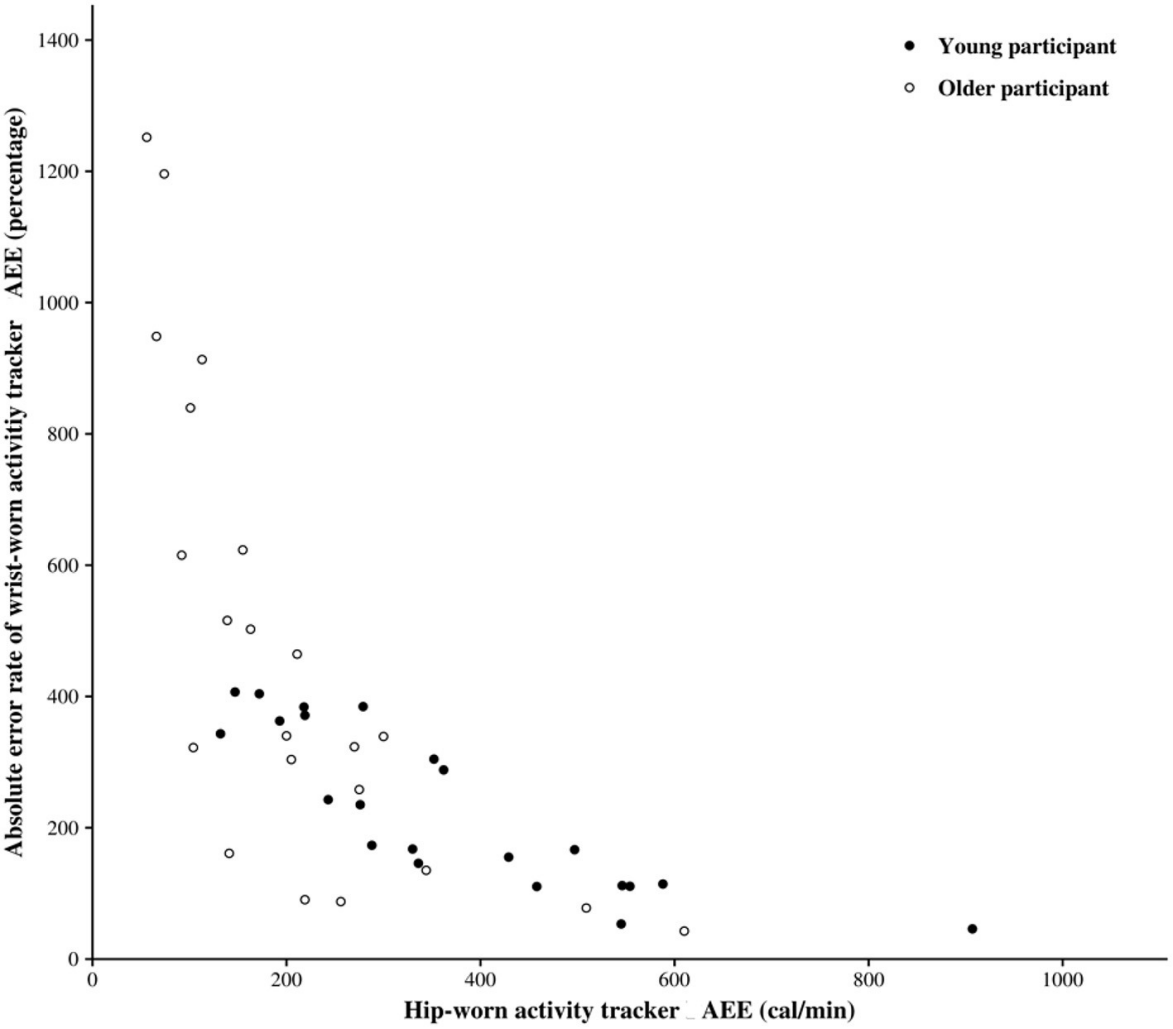
Absolute error rate (AER) between active energy expenditure (AEE) provided by hip-worn and wrist-worn activity tracker.

Figure 4 shows the absolute error rate (AER) of the AEE provided by the wrist-worn AT in comparison to the AEE provided by the hip-worn AT. The AER and the hip-worn AEE had a strong negative correlation in the overall population (spearman's rho = 0.84, *p* = 0.001), in young participants (spearman's rho = 0.83, *p* = 0.001), and in older adults (spearman's rho = 0.87, *p* = 0.001). In other words, the error in AEE provided by the wrist-worn AT tends to decrease according to energy expended as evaluated by the hip worn AT. Finally, a significant difference was observed between AER of older and young participants (*p* < 0.05).

## Discussion

The objective of this study was to compare the AER of AEE measured by the wrist-worn and the hip-worn ActiGraph GT3X over a 24-h period in free-living conditions in young and older adults. Our results show that AEE was overestimated when measured by the wrist-worn AT (3.2 times higher than AEE provided by the hip-worn AT). Furthermore, we found a significant negative correlation between AER and AEE provided by the hip-worn AT in the overall population. We also report an effect of age on the AER with a lower AER for young participants. Finally, the AER tends to decrease when the AEE increases.

### AEE as a Function of Location of the ActiGraph GT3X+ on the Body

When the ActiGraph GT3X was placed at the hip, the AEE generated less error between the two body locations (hip vs. wrist). The conventional AT was made to be worn around the hips, with a belt or waistband (33). According to the systematic review of Migueles et al. (17), the accuracy of the AEE measurement increases when the AT is worn on the hip compared to the wrist. This increased precision could be explained by the fact that an AT worn on the hip is closer to the body's center of mass, making detection of the entire body's acceleration easier. In addition, other factors may be involved:

- The AT worn on the wrist records all forearm accelerations. Throughout the day, an individual is exposed to a variety of tasks that could involve the use of the upper limbs only. While seated, for example, one can actively move one's hands while eating, speaking, or interacting with a screen, among other activities (34).
- Hildebrand et al. (21) showed that accelerometer output provided by the GT3X+ worn at the wrist was 200% higher than the AT worn at the hip during step activity. Mandigout et al. (31) found that step counts provided by the wrist-worn AT were overestimated compared to the results provided by the hip-worn AT. This leads to an overestimation of energy expenditure. Similarly, Ceaser (35) proved that EE was overestimated by the AT worn at the wrist in most walking activities and activities involving arm movements.

### AER as a Function of the Age of the Participants

Our results show that AER is more important when AEE is low. More interestingly, we found a higher AER in older participants.

The decrease in AT accuracy reported in the literature when recording slow activities is one of the criteria that potentially explain our findings (36). Indeed, multiple research show that only a few AT can record motion slower than 1 m.s−1 (37). In fact, older persons (60+) have been observed to choose a walking speed of 1.18 m.s−1 (±0.17 m.s−1) (38) that can increase to 1.34 m.s−1 (±0.21 m.s−1) in healthy older adults (39). Webber and St. John (14) conducted a comparative study (ActiGraph vs. Stepwatch, Hip vs. Ankle) in 38 elderly rehabilitation patients (83.2 ± 7.1 years old), walking at a comfortable pace (0.4 m.s−1) during a hallway walk. Elderly populations and people with motor impairments commonly report speeds <1 m.s−1. In the study of Weber and St. John (14), the AER was low (<3%) for slow walking speeds and did not differ substantially between the StepWatch and the Actigraph GT3X+ (worn at the ankle). However, error values were larger (19–97%) when the Actigraph GT3X+ was worn at the hip during a hallway walk. Our findings suggest that a wrist-worn activity monitor may overestimate low-speed activities while underestimating high-speed activities. Wrist kinematics, according to Aziz et al. (34), may represent a small portion of total body movements during walking (particularly when walking with little arm swing) and a bigger portion during other sedentary activities, such as merely moving hands while sitting.

Our results could also be explained by the brief time spent in moderate activity under 24 h by the older participants. Actually, Crouter et al. (40) founded that the Actigraph tended to overestimate walking and sedentary activities and underestimate most other activities in healthy adults. In other words, the error will be greater for activity with low intensity which could be the case of older adults in our study.

### Practical Application

The present findings evidenced that two identical ActiGraph GT3Xs placed at the hip or wrist provide an AER in real life situations. This error is greater when AEE is low. More interestingly, our findings suggest that this AER depends on several factors. Our findings suggest that the accuracy of the AT, which is directly dependent on the technology and processing algorithm, should be addressed differently for young and old participants, as well as patients with motor impairments. As a result, it appears that precisely identifying the target group and the type of activity desired is critical. Furthermore, scientific evidence clearly demonstrates the need of using the same AT on a regular basis. Currently, firms are working on this. There are many various models on the market, and the EE obtained for a given activity can vary greatly from one AT to another (33). Additionally, the location of the AT on the body and the wearer's age appear to be even more important factors.

### Contribution of This Manuscript to the Field of Physical Activity

The publication of this manuscript aims to complete the conclusion of our previous article interested in step count (31). We found that the step count differs according to the position of the AT with a more important error in the older participants. In the present article, we are interested in the EE which is one of the main parameters to assess the intensity of PA. It is also the only parameter for which we have recommendations for PA. Taken together, results of our two complementary studies, we found that there is a less dispersion of AER's step count (from <10% to more than 350%) than the dispersion of AER's AEE (from 43 to 1,252%). These findings show that using AEE or step count as an outcome to assess physical activity should be treated with caution. We may also suggest the same caution for implementation of other activity trackers which are now widely used in PA assessment.

### Limitations of the Study

Our study's small sample size (*n* = 44) may be a limitation. However, our findings are supported by numerous published studies and serve as a valuable supplement to the ActiGraph GT3X's wrist and hip applications (18, 21, 31, 41). Furthermore, since the gold-standard solution for evaluating the AEE in free-living situations (doubly labeled water) is expensive and difficult to generalize across a population, our research does not allow us to pretend which ActiGraph GT3X provides the most precise values, i.e., the closest to reality. Further research is needed to determine the best AT placement on the bodyand the most common positions in which these sensors are used by consumers in free-living situations. In light of our findings, it appears that in order to reach this goal, it will be required to carefully analyze the position of the AT, the age of the users, and their lifestyle habits.

## Conclusion

In young and elderly adults in free-living conditions, wearing the AT at the wrist may result in an overestimated AEE when compared to wearing the identical AT model at the hip. On the one hand, a difference appeared according to the age of the participants. On the other hand, it appears that the gap between the two body locations has decreased as AEE has increased whatever the age. The amount of PA in free-living situations assessed using EE remains uncertain and imprecise. For all populations (young, old, healthy or patients), more effort is needed to increase the quality of activity tracker-based EE assessment in free-living situations.

## Data Availability Statement

The datasets generated for this study are available on request to the corresponding author.

## Ethics Statement

The protocol was approved by the Comité d'Ethique pour les Recherches Non Interventionnelles (Ethics Committee for Non-Interventional Research) (CERNI) of the Grenoble-Alpes University, France. All subjects gave written informed consent in accordance with the Declaration of Helsinki.

## Author Contributions

JL and SM: conceptualization. JL and LR: methodology. SM: validation, supervision, and project administration. TA and NV: formal analysis. JL: investigation. AG and LR: writing—original draft preparation. NV and SM: writing—review and editing. NV: funding acquisition. All authors have read and agreed to the published version of the manuscript.

## Conflict of Interest

The authors declare that the research was conducted in the absence of any commercial or financial relationships that could be construed as a potential conflict of interest.

## Publisher's Note

All claims expressed in this article are solely those of the authors and do not necessarily represent those of their affiliated organizations, or those of the publisher, the editors and the reviewers. Any product that may be evaluated in this article, or claim that may be made by its manufacturer, is not guaranteed or endorsed by the publisher.
